# Survey in ruminants from Rwanda revealed high diversity and prevalence of extended-spectrum cephalosporin-resistant Enterobacterales

**DOI:** 10.1186/s12917-024-04359-3

**Published:** 2024-11-19

**Authors:** Emmanuel Irimaso, Helga Keinprecht, Michael P. Szostak, Adriana Cabal Rosel, Beatrix Stessl, Amelie Desvars-Larrive, Christophe Ntakirutimana, Otto W. Fischer, Thomas Wittek, Elke Müller, Andrea T. Feßler, Sascha D. Braun, Stefan Schwarz, Stefan Monecke, Ralf Ehricht, Joachim Spergser, Werner Ruppitsch, Igor Loncaric

**Affiliations:** 1 School of Veterinary Medicine- CAVM, University of Rwanda, Nyagatare, Rwanda; 2 Institute of Microbiology, University of Veterinary Medicine Vienna, Vienna, Austria; 3 New Vision Veterinary Hospital (NVVH) Northern Province, Musanze District, Rwaza Sector, Musanze, Rwanda; 4 Austrian Agency for Health and Food Safety (AGES), Institute of Medical Microbiology and Hygiene, Vienna, Austria; 5 Unit of Food Microbiology, Institute of Food Safety, Food Technology and Veterinary Public Health, University of Veterinary Medicine Vienna, Vienna, Austria; 6 Unit of Veterinary Public Health and Epidemiology, Institute of Food Safety, Food Technology and Veterinary Public Health, University of Veterinary Medicine Vienna, Vienna, Austria; 7 Complexity Science Hub Vienna, Vienna, Austria; 8 Clinical Unit of Ruminant Medicine, University of Veterinary Medicine, Vienna, Austria; 9 Leibniz Institute of Photonic Technology (IPHT), Jena, Germany; 10 InfectoGnostics Research Campus, Jena, Germany; 11 Institute of Microbiology and Epizootics, Centre of Infection Medicine, Department of Veterinary Medicine, Freie Universität Berlin, Berlin, Germany; 12 Department of Veterinary Medicine, Veterinary Centre for Resistance Research (TZR), Freie Universität Berlin, Berlin, Germany; 13 Institut für Medizinische Mikrobiologie und Hygiene, Universitätsklinik Dresden, Dresden, Germany; 14 Institute of Physical Chemistry, Friedrich Schiller University Jena, Jena, Germany

**Keywords:** ESBL, Whole-genome sequencing, Rwanda, “One Health”, Antimicrobial resistance

## Abstract

**Background:**

Antimicrobial resistance (AMR) in Enterobacterales constitutes a significant threat to the health of both humans and animals and a socioeconomic problem. Enterobacterales, mainly *Escherichia coli*, carrying β-lactamases has become one of the main indicators to estimate the burden of AMR in animals within “One Health” approach.

**Objectives:**

To assess the presence of extended-spectrum cephalosporin-resistant Enterobacterales associated with ruminants (cattle, sheep, goats) habituated in all five provinces of Rwanda and to perform in depth characterization of isolates.

**Methods:**

We screened 454 rectal swabs from 203 cows, 170 goats, and 81 sheep and selective isolation of extended-spectrum cephalosporin-resistant Enterobacterales was conducted. Isolates were identified as a members of the order Enterobacterales by MALDI-TOF MS and further characterized by susceptibility testing and by whole-genome sequencing.

**Results:**

Out of the 454 samples, 64 extended-spectrum cephalosporin-resistant Enterobacterales were isolated from 58 animals. Isolates belonged to seven bacterial species and were identified as *Escherichia coli* (*n* = 54), *Enterobacter bugandensis* (*n* = 4), *Enterobacter mori* (*n* = 2), *Klebsiella pneumoniae* (*n* = 2), *Enterobacter dykesii* (*n* = 1), and *Citrobacter freundii* (*n* = 1). All isolates displayed an Extended-spectrum β-lactamases (ESBL) phenotype, with exception of *Citrobacter freundii* isolate displayed both an ESBL and AmpC phenotype. In addition, all *Enterobacter* isolates were identified as stably de-repressed AmpC-producers. ESBLs genes, *bla*_CTX−M−15_ was predominant. Resistance to tetracycline and *tet*(A) was most frequently observed among non-β-lactam resistance. Forty-eight isolates displayed multidrug-resistance phenotypes. A shiga toxin-producing *E. coli* and an enterotoxigenic *E. coli* isolate were observed. Genome comparisons revealed thirty-five *E. coli* sequence types (ST) (ST10, ST307 being predominate).

**Conclusions:**

Considering the high proximity between ruminants and humans in Rwanda, the dissemination of antimicrobial drug resistance highlights the public health threats and requires the joint and multisectoral action of human and veterinary medicine, at human-animal-environment interfaces. Therefore, it is important to establish national and global “One Health” surveillance programs of AMR to tackle the antibiotic-resistant crisis in human and veterinary medicine.

**Supplementary Information:**

The online version contains supplementary material available at 10.1186/s12917-024-04359-3.

## Background

The different β-lactam antibiotics are most extensively used for the treatment of bacterial infections in human and veterinary medicine [1, 2]. Antimicrobial resistance (AMR) in bacteria constitutes a significant threat to the health of both humans and animals and a socioeconomic problem [3]. For multidrug-resistant members of Enterobacterales – and primarily β-lactamase producers –limited therapeutic options exist [4]. The most common β-lactamases are SHV, TEM and CTX-M variants of extended-spectrum β-lactamases (ESBLs) [5, 6]. ESBLs confer resistance to penicillins, I., II., and III. generation cephalosporins and monobactams [7], but can inhibited by β-lactamase inhibitors [5]. ESBL-producing Enterobacterales are associated with a high burden in terms of morbidity and mortality [8]. Therefore, the World Health Organization (WHO) listed ESBL-producing Enterobacterales among others in the most critical group according to the urgency of the need for the development of new antibiotics [9]. Enterobacterales harboring ESBL genes have been isolated across continents from humans, domestic and wild animals, and the environment [3, 7, 10].

Rwanda recognized the challenging situation of AMR as a significant public health problem and a threat to its unique population of mountain gorillas (*Gorilla beringei* ssp. *beringei*) and other endangered wildlife. In consequence, based on five objectives of the Global Action Plan of the WHO [11], Rwanda developed the National Action Plan on Antimicrobial Resistance [12]. Nevertheless, information on AMR in bacteria associated with human infections and domestic animals remains scarce.

The present study aimed to determine the presence of extended-spectrum cephalosporin-resistant Enterobacterales associated with ruminants (cattle, sheep, goats) located in all five provinces of Rwanda and to characterize these isolates.

The Rwandan economy relies mainly on the agriculture sector, where subsistence farming is still predominant. Cattle farming has a long tradition and is anchored in the history of the country [13]. Farmers have free access (without prescription) to veterinary drugs, like antibiotics and most medicines, while on the other hand, microbiological diagnostic options in veterinary medicine are limited [12].

## Materials and methods

### Sample collection and identification of Enterobacterales

To determine the presence of enteric colonization by extended-spectrum cephalosporin-resistant Enterobacterales, a total of 454 rectal swabs from 203 cows, 170 goats, and 81 sheep, were sampled between September until December 2021. Animals were selected based on owner´s willingness to participate to the study and only if they did not receive any antimicrobial treatment in the past six months.

The study was discussed, and the rectal swabbing was approved by the Research Screening and Ethical Clearance Committee of the College of Agriculture, Animal Sciences and Veterinary Medicine, University of Rwanda (003/2021/DRI).

In the microbiological laboratory at the New Vision Veterinary Hospital in Rwanda (https://nvvh.rw/) each rectal sample was incubated at 37 °C overnight in buffered peptone water (Merck), with cefotaxime (1 mg/L) and then cultivated at 37 °C overnight on MacConkey agar (Rapid Labs, Colchester, United Kingdom) supplemented with cefotaxime (1 mg/L) (MacCTX). After incubation on MacCTX, one colony representing a distinct colony morphotype (e.g. shape, size, color, mucoid yes/no, lactose fermentation was subcultured on the same medium and cryo-conserved. For species identification and further in-depth characterization, isolates were sent to the Institute of Microbiology, University of Veterinary Medicine Vienna, Austria. Isolates were identified to the species level by matrix-assisted laser desorption ionization-time-of-flight mass spectrometry (MALDI-TOF MS) (Bruker Daltonik, Bremen, Germany). Only isolates that belonged to the order Enterobacterales and that could grow on MacCTX were selected for further characterization.

## Susceptibility testing

Antimicrobial susceptibility testing was performed by agar disk diffusion according to the Clinical & Laboratory Standards Institute (CLSI) standards [14]. *Escherichia coli* ATCC^®^ 25922 served as the quality control. Disks containing the following antimicrobial agents were used: cefotaxime (30 µg), ceftazidime (30 µg), cefoxitin (30 µg), meropenem (10 µg), gentamicin (10 µg), tobramycin (10 µg), amikacin (30 µg), ciprofloxacin (5 µg), trimethoprim-sulfamethoxazole (1.25/23.75 µg), tetracycline (30 µg), chloramphenicol (30 µg), fosfomycin (200 µg), and nitrofurantoin (300 µg) (Becton Dickinson, Heidelberg, Germany). AmpC-hyperproducing isolates (stably de-repressed) of *Enterobacter* spp. and *Citrobacter* spp. were defined as previously described [15].

## Molecular analyses

Isolates were analyzed by whole-genome sequencing (WGS). Isolation of bacterial DNA, library preparation, and paired-end-sequencing using NextSeq2000 platform were performed as described previously [16]. *De novo* assembly of the raw reads was performed using SPAdes v.3.9.0 [17], and typing was performed with SeqSphere + software (Ridom, Münster, Germany). The species allocation was confirmed by JSpecies workspace using the ANIb (average nucleotide identity via BLAST) analysis tool [18].

*E. coli* phylotypes were determined based on WGS by the Clermont Typing tool (http://clermontyping.iame-research.center/, accessed on 2023 May 30) [19, 20]. Clonal relatedness of *E. coli* isolates was assessed by two-locus sequence typing of combined data of *fumC* and *fimH* sequences using CHTyper [21] hosted at the Center for Genomic Epidemiology (https://cge.cbs.dtu.dk/services/chtyper/, accessed on 2023 May 30). Sequence types (STs) of *E. coli* were determined using the *Escherichia/Shigella* database hosted on EnteroBase [22]. SeqSphere + software (Ridom, Münster, Germany) was used for core-genome Multilocus sequence typing (cgMLST) analysis of *E. coli* and *Klebsiella pneumoniae* isolates. The same software was used to predict serogenotypes of *E. coli*. STs of *K. pneumoniae* were determined using the *K. pneumoniae* species complex database hosted at the Institute Pasteur (https://bigsdb.pasteur.fr/klebsiella/*).* The same database was used to examine the presence of virulence-associated genes (VAGs). STs of *Enterobacter* (*En.*) *cloacae* complex and *Citrobacter* (*C.*) *freundii* were assigned using databases hosted at PubMLST (*Enterobacter cloacae* complex: https://pubmlst.org/organisms/enterobacter-cloacae, *Citrobacter* sp.: https://pubmlst.org/organisms/citrobacter-spp [23]. ABRicate [24] v1.0.0 was used to identify antimicrobial resistance genes using ResFinder 4.1 [25], VAGs were identified using Virulence Factor Database [26], and virulence-associated genes in *E. coli* using VirulenceFinder 2.0 [27]. The presence of plasmids was assessed using PlasmidFinder 2.1 [28]. The beta-lactamase database (BLDB) was used to classify some of β-lactamase genes [29]. Probability prediction of the location of a given *bla* resistance gene in Enterobacterales was achieved by applying mlplasmids trained on *E. coli* or *Klebsiella* [30]. Posterior probability scores (ppp) > 0.7 and a minimum contig length of 700 bp indicate that a given contig is plasmid-derived. For *bla* genes with ppp scores below 0.7, Basic Local Alignment Search Tool (BLAST) searches with the respective contig sequence were performed against all Enterobacterales plasmids at National Center for Biotechnology Information (NCBI). The most promising plasmid entries were then used as backbones in mapping all plasmid contigs of a given isolat using GENEious Prime software 2022.2.2. A plasmid location was assumed only if the combined BLAST/GENEious analysis was able to identify a published plasmid which could be reconstructed with > 90% identity. Additional gene prediction and annotation were performed using the Bioinformatics Resource Center PATRIC [31].

The genomes of isolates subjected to WGS were deposited under PRJNA950594 in the NCBI BioProject database.

## Statistical analyses and data visualization

Statistical analyses and visualizations were computed in R 4.2.3 [32]. Descriptive statistics were used to characterize the study population and describe the results. The 95% confidence interval (95%CI) of the prevalence was calculated using the R package epiR [33] A generalized linear model (GLM) with binomial distribution and logit function was applied to identify variables that may explain the presence/absence of extended-spectrum cephalosporin-resistant Enterobacterales in animals (presence was defined as the carriage of at least one resistant isolate). The impact of the following explanatory variables was assessed: *province* (Northern, Eastern, Western, Southern, Kigali City), *animal species* (bovine, ovine, caprine,, with ovine used as reference category), and *sex* (female, male, with female used as reference category). The best-fitted model (outlining the most important predictors and their effects on the outcome variable) was identified using a stepwise backward selection based on the Akaike Information Criterion. Estimates were transformed to odds ratios (OR). Moreover, difference between prevalences was analyzed by using the 2-tailed 2-proportion z-test.

## Results

### Isolation of cefotaxime resistant isolates, phenotypic resistance and species identification

A large majority of the sampled animals were female (95.4%). Animals´ age ranged between 0.3 and 11 months (29% of the data on age was unavailable). Overall, 166 swabs were collected in the Northern Province (71 bovines/51 caprines/44 ovines), 127 in the Eastern Province (66/57/4), 103 in the Western Province (38/49/16), 55 in the Southern Province (25/13/17), and three in Kigali City (3/0/0). Out of the 454 samples, 64 extended-spectrum cephalosporin-resistant Enterobacterales were isolated from 58 animals (prevalence = 12.8%; 95%CI = [9.8–16.2]), including 33 bovines (16.3%; 95%CI = [11.5–22.1]), 20 caprines (11.8%; 95%CI = [7.3–17.6]), and 5 ovines (6.2%; 95%CI = [2.0–13.8]). The prevalence in females was 12.9% (95%CI = [9.9–16.5]) and was statistically not significant (*p* = 0.9) from 9.5% in males (95%CI = [1.2–30.4]).

Isolates belonged to seven bacterial species and were identified as *E. coli* (*n* = 54), *En. bugandensis* (*n* = 4), *En. mori* (*n* = 2), *K. pneumoniae* (*n* = 2), *En. dykesii* (*n* = 1), and *C. freundii* (*n* = 1) (Additional file 1: Table S1). All *E. coli* and *K. pneumoniae* isolates displayed an ESBL phenotype, whereas the *C. freundii* isolate displayed both an ESBL and AmpC phenotype. In addition, all *Enterobacter* isolates were identified as stably de-repressed AmpC-producers.

## Molecular characterization

Among genes associated with resistance to β-lactams, *bla*_CTX−M−15_ was identified in 47 isolates, followed by *bla*_TEM−1_ (*n* = 35). These two β-lactamase genes were observed together in 33 isolates. Among other *bla*_CTX−M_ alleles, *bla*_CTX−M−14_ (*n* = 3), *bla*_CTX−M−137_ (*n* = 2), and single *bla*_CTX−M−121_ and *bla*_CTX−M−27_ were identified in *E. coli* isolates. The *bla*_SHV_ genes were less frequently detected, *bla*_SHV−208_ genes were identified together with *bla*_CTX−M−15_ and *bla*_TEM−1_ in two *K. pneumoniae* isolates. The ESBL gene *bla*_SHV−12_ was detected in an *E. coli* isolate. The oxacillinase gene *bla*_OXA−1_ was observed in three *E. coli* isolates. The genes belonging to *bla*_EC_ family belonging to class C β-lactamases (cephalosporinase) were observed in 53 isolates. In addition to one not further specified *bla*_EC_ gene (designated *bla*_EC−NT_) and the following *bla*_EC_ genes were identified *bla*_EC−1_ (*n* = 3), *bla*_EC−4_ (*n* = 2), *bla*_EC−8_ (*n* = 10), *bla*_EC−13_ (*n* = 8), *bla*_EC−15_ (*n* = 9), *bla*_EC−18_ (*n* = 15), *bla*_EC−1502_ (*n* = 2), *bla*_EC−2021_ (*n* = 1), and *bla*_EC−2045_ (*n* = 2) (Additional file 1: Table S1).

**Fig. 1:**
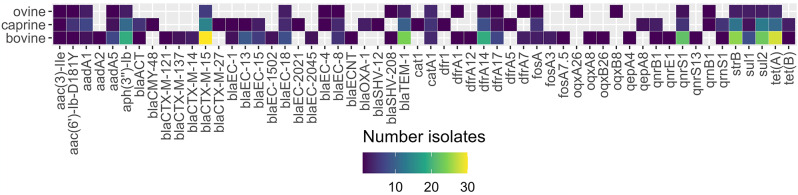
Sankey graph showing the animal hosts, the isolated antimicrobial resistant Enterobacterales (*n* = 64), and their corresponding genotypes, Rwanda, September-December 2021

Among non-β-lactam resistance, resistance to tetracycline (*n* = 45; 70.3%), trimethoprim-sulfamethoxazole (*n* = 41; 64.1%), fosfomycin (*n* = 13; 20.3%), chloramphenicol (*n* = 11, 17. 2%), tobramycin (*n* = 8, 12.5%), ciprofloxacin (*n* = 6, 9.4%), gentamicin (*n* = 3, 4.7%), and nitrofurantoin (*n* = 1, 1.6%) was detected. Visualization of genotypes per isolated bacterial species is shown in Fig. 1 The most prevalent non-β-lactamase genes detected were *tet*(A) (*n* = 41), *sul2* (*n* = 38), *strB* (also known as *aph* [6]*-Id*) (*n* = 38), *aph(3’’)-Ib* (*n* = 33), *dfrA14* (*n* = 29), and *qnrS1* (*n* = 28). Forty-eight isolates displayed multidrug-resistance phenotypes [34].

Among the ciprofloxacin-resistant isolates, significant mutations in the quinolone resistance-determining regions (QRDR) of *gyrA*, *parC* and *parE* were observed. The mutations led to amino acid substitutions at positions 83 (Ser→Leu) and 87 (Asp→Asn) in GyrA in five isolates and to one exchange at position 83 (Ser→Leu) in one isolate. ParC was altered at position 80 (Ser→Ile) in all six ciprofloxacin-resistant isolates as well as at position 458 (Ser→Ala) in two isolates, at position 458 (Ser→Thr) in one isolate, and at position 416 (Leu→Phe) in another isolate (Additional file 1: Table S1).

Genes associated with biocide resistance were detected in 23 isolates, namely *qacE* (*n* = 13), *sitABCD* (*n* = 6), *oqxA* (*n* = 2), and *oqxB* (*n* = 2), which are associated with the decreased susceptibility to benzalkonium chloride, chlorhexidine, and hydrogen peroxide (Additional file 1: Table S1).

Different virulence-associated genes were observed in *E. coli* isolates, with the tellurite resistance gene *terC* (*n* = 54) and the *fimH* gene encoding the mannose-binding type 1 pilus tip protein FimH (*n* = 49) being predominant. Among *E. coli* pathotypes, one caprine Shiga toxin-producing *E. coli* (STEC) carrying *stx1c* and *stx2b*, one bovine enterotoxigenic *E. coli* (ETEC) isolate carrying *astA* (coding for heat-stable enterotoxin EAST-1) and *eltIAB* (coding for heat-labile enterotoxin LTIh) were observed. Among *K. pneumoniae* virulence factors, type 3 fimbriae encoded by the *mrk* operon and genes coding for yersiniabactin (*ybt*) were detected in all *K. pneumoniae* isolates (Additional file 1: Table S1).

*E. coli* phylotyping revealed that the most predominant phylogroups were A and B1 (each *n* = 20), followed by D (*n* = 9), C (*n* = 2), B1 mash E (*n* = 2) and cladeI (*n* = 1). The *fumC* and *fimH* (CH) typing divided the *E. coli* isolates into 38 distinct CH clonotypes, wherefrom CH clonotype 29–87 was predominant (*n* = 4). Two novel *fimH* alleles were detected: fimH3351 (isolate 511398-22) and fimH3352 (isolate 511421-22).

**Fig. 2:**
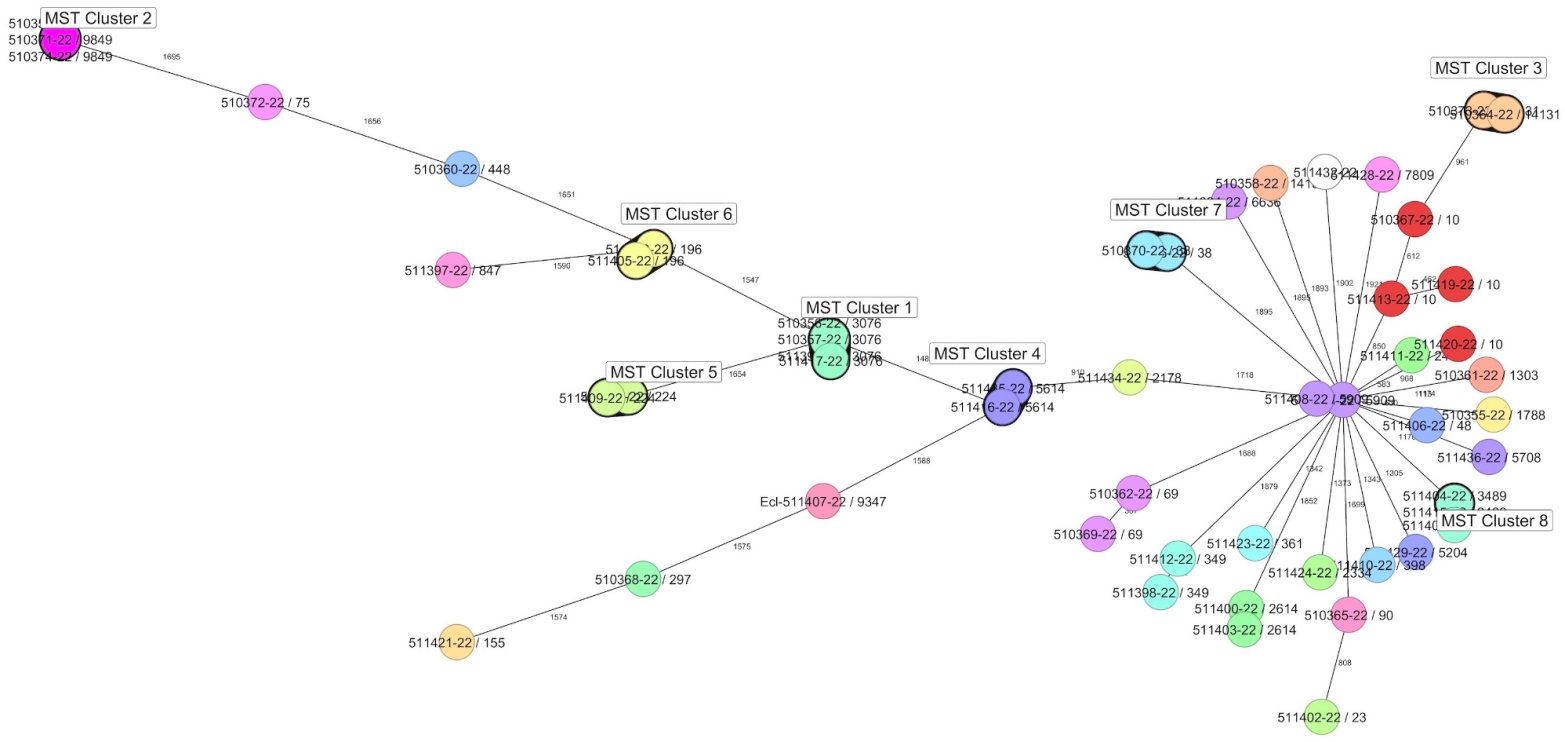
Minimum spanning tree comparing *E. coli* isolates. Easch circle represents isolates with an allelic profile based on the cgMLST which consists out of 2513 alleles. Numbers correspond to the allelic differences between isolates; isolates with closely related genotypes are in bolded circles and marked as clusters. Isolates were labeled according to isolate ID (/) classical MLST

Overall, 44 STs were evidenced that showed a heterogeneous spatial distribution over the Rwandan provinces. Thirty-five *E. coli* sequence types (ST) (based on seven loci MLST extracted from WGS data) were detected: ST10, ST3076 (each *n* = 4), ST9849, ST3489 (each *n* = 3), ST69, ST5614, ST38, ST224, ST5909, ST196, ST2614, ST14131, ST349 (each *n* = 2) and single isolates of ST2334, ST14130, ST90, ST297, ST23, ST155, ST7809, ST1788, ST2465, ST13718, ST5708, ST361, ST6636, ST2178, ST75, ST398, ST847, ST448, ST9347, ST48, ST1303, and ST5204, wherefrom ST13718, ST14130, and ST14131 are novel STs. The analysis of WGS data revealed that ten isolates could be assigned to WGS-predicted serotypes (somatic O and flagellar H antigens) O1:H9, O5:H11, O8:H9 (*n* = 3), O8:H53, O15:H18, O101:H10 (*n* = 2), and O131:H45. In the remaining isolates, the O or H type could not be assigned (Additional file 1: Table S1). WGS-based cgMLST revealed eight clusters comprising four isolates (Cluster 1, ST3076), three isolates (Cluster 2, ST9849), two isolates (Cluster 3, ST14131; Cluster 4, ST38; Cluster 5, ST196; Cluster 6, ST224; Cluster 7, ST5614, and Cluster 8, ST3489) (Fig. 2). All other isolates were singletons. Out of seven *Enterobacter* spp. isolates, six were novel, ST2013 (*En. dykesii*), ST2014, ST2015, ST2017 (*En. bugadensis*), ST2016 and ST2088 (*En. mori*). ST733 (*En. bugadensis*) was an already established ST. Other STs obtained were ST610 (both *K. pneumoniae* isolates) and *C. freundii* ST163 (Additional file 1: Table S1).

The presence of the plasmid replicons among β-lactamase gene carrying isolates was analyzed by PlasmidFinder and revealed that ten different plasmid replicons could be detected, with IncB/O/K/Z being predominant. The mlplasmids analyses and BLAST searches predicted that the majority of the β-lactamase genes might be plasmid-encoded and the majority of the other resistance genes appeared to be chromosomally encoded. In the present study, it was observed that some *bla* genes were carried on plasmids, including *bla*_CTX−M−15_ by IncB/O/K/Z, IncFIB(AP001918), IncFIB (H89-PhagePlasmid), IncFII, IncFII(K), IncHI2A, and IncQ1IncY plasmids, *bla*_CTX−M−27_ by IncFII (pRSB107) plasmid, *bla*_TEM−1B_ by IncFII (pHN7A8), IncI1-I (Alpha), and IncY plasmids, *bla*_SHV−12_ on IncY plasmid. The full list of AMR genes and their predicted plasmid probability are shown in Additional file 2: Table S2. Genome data are shown in additional file 3: Table S3.

## Occurrence of extended-spectrum cephalosporin-resistant Enterobacterales among different animals

The best GLM model selected according to the AIC values included only animal species as explanatory variable. The odds of extended-spectrum cephalosporin-resistant Enterobacterales occurrence was nearly threefold greater for bovines compared to ovines (OR = 2.95, 95% IC = [1.20–8.88]). No significant difference was observed between ovines and caprines (OR = 2.03, 95% CI = [0.79–6.28]).

## Discussion

The present study aimed at investigating phenotypic and genotypic characteristics of extended-spectrum cephalosporin-resistant Enterobacterales isolates obtained from rectal swabs of ruminants (cattle, sheep, goats) from all five provinces in Rwanda. To the best of authors knowledge, the present study is the first study dealing with in-depth analyses of extended-spectrum cephalosporin resistance in Enterobacterales associated with ruminants in Rwanda.

In this study, the extended-spectrum cephalosporin resistance in Enterobacterales was detected at a frequency of 12.8% (58/454) in rectal samples of Rwandan ruminant livestock species. This is of interest because third- or fourth-generation cephalosporins are either not used for the treatment of domestic animals in Rwanda yet [35] and rarely used for the treatment of wild animals [36]. In addition, none of the animals examined during the present study was associated with known risk factors for the infection and/or colonization with AMR, such as recent veterinary health care (during the last six months) or treatment with antimicrobial substances in the preceding six months.

Antimicrobials are usually not used to treat goats and sheep in Rwanda. Even though the occurrence of extended-spectrum cephalosporin-resistant Enterobacterales in caprine and ovine samples was less frequent than in bovine, this is of particular concern. In Rwanda, small ruminants usually live in close proximity with their owners, often sharing the same premises with humans during the night. Interestingly, caprine ESBL-producing *E. coli* isolates were obtained solely from the Western provinces. Five out of 13 STs observed among caprine *E. coli* isolates have already been isolated from humans in Rwanda (ST10, ST155, ST361, ST38, and ST69) and from an animal of unknown species (ST38) [37]. Among them, members of ST10, ST38, ST69, ST155 belong to important pandemic clonal lineages, which are often multidrug-resistant and usually associated with numerous ESBL genes [38, 39]. With the exception of one *E. coli* harboring *bla*_CTX−M−27_ belonging to the ST90, and an *E. coli* isolate harboring *bla*_SHV−12_, all other caprine isolates carried *bla*_CTX−M−15_. CTX-M-15, a subtype in the CTX-M-1 group, is a globally disseminated β-lactamase and was detected in *E. coli* isolates associated with humans and animals [40]. The *bla*_CTX−M−15_ gene was also the most predominant β-lactamase gene among ESBL *E. coli* isolates in Rwanda from hospitalized patients, caregivers, and the community [37] as well as from flies from a tertiary hospital [41]. CTX-M-27, together with CTX-M-15 and CTX-M-14, are dominant among Enterobacterales worldwide [42]. While *bla*_CTX−M−15_ and *bla*_CTX−M−27_ positive *E. coli* have already been reported [37], to our knowledge, this is the first report of *E. coli* harboring *bla*_SHV−12_, in Rwanda. SHV-12 is an important ESBL associated with nosocomial and community-acquired infections in several countries and has also been reported in *E. coli* associated with domestic and wild animals [7, 43, 44]. Besides resistance-associated genes, genes coding for virulence factors were observed in all isolates. In the *bla*_CTX−M−15_-carrying isolate belonging to ST75, the Shiga toxin genes were observed. ST75 has already been reported in numerous countries in Asia, Europe, North America, and Oceania. Still, there are no ST75 entries from Africa neither in the Enterobase *Escherichia/Shigella* Database (https://enterobase.warwick.ac.uk/species/index/ecoli, last accessed 2023 May 30) nor in the *E. coli* PubMLST Database (https://pubmlst.org/organisms/escherichia-spp), last accessed 2023 May 30). In a multi-resistant *bla*_CTX−M−15_-*E. coli* ST297, genes coding for F17 fimbriae, the heat-labile enterotoxin LTII (*eltIIAB*), and other VAGs were observed. The fimbrial adhesin F17 is associated with ETEC in calves [45].

Among the characterized ovine isolates, an atypical enteropathogenic *E. coli* (aEPEC) belonging to ST2178, a common clone in Africa (https://enterobase.warwick.ac.uk/species/index/ecoli, last accessed 2023 May 30) was observed. This isolate carried the *astA* gene (coding for heat-stable enterotoxin EAST-1) and other virulence-associated genes (VAGs). EAST-1 has been associated with diarrheal disease in humans and numerous animal species, mainly cattle and swine [46].

Since cattle play such an important role in the lives of the people in Rwanda, the detection of multidrug-resistant *E. coli* isolates is of great importance, especially the very close proximity between humans and cattle induces a risk of animal-to-human transmission of AMR. Six out of 22 different STs detected among bovine ESBL *E. coli* isolates (ST10, ST196, ST224, ST48, ST448, and ST69) have previously been observed among human isolates [37], whereas ST10 and ST69 are well-known pandemic lineages [38, 39]. The *bla*_CTX−M−15_ was the most predominant ESBL gene among bovine isolates. Furthermore, *bla*_CTX−M−14_, *bla*_CTX−M−121_, and *bla*_CTX−M−137_ were observed. CTX-M-137, a hybrid of CTX-M-14 [47] and CTX-M-121 [48], both CTX-M-9 group-like β-lactamases, is rarely reported among *E. coli* isolates, and to our knowledge, there is no published data reporting the presence of *E. coli* harboring this β-lactamase gene in Rwanda. An *astA* gene accompanied by other VAGs was observed in three STs, ST10, ST349, and ST847. Even though EAST-1 was associated with diseased humans and animals, the virulence capacity and pathogenesis of the EAST-1 toxin remains uncertain and disputed [46].

The predominant *E. coli* phylotypes in the present study were groups A and B1. Both phylogroups are described as being widespread among human commensal *E. coli* isolates [49]. Phylogroup D was common in our study (*n* = 9). It was previously described to be associated with *E. coli* isolates associated with extra-intestinal infections in humans [50].

Based on cgMLST analysis eight clusters and 35 singletons were observed. Two clusters (cluster 3 (ST14131) and cluster 6 (ST196)) comprise two isolates from the same farm and originated from the same species (bovine or caprine). All other clusters contain isolates that originated (a) from the same sector and the same animal species but different farms (cluster 7 (ST38)), (b) from the same province and the same animal species but different sectors (cluster 1 (ST3076)), (c) from different provinces but the same species (clusters 5 (ST224) and 8 (ST3489)), and finally (d) from different provinces and different animals species (cluster 4 (ST5614). Interestingly, from one rectal sample two *E. coli* belonging to different clusters could be detected (strain IDs 511405-22 and 511415-22). These findings suggested clonal distribution and interspecies transmission of extended-spectrum cephalosporin-resistant *E. coli* in examined animals throughout Rwanda.

Two CTX-M-15 positive *K. pneumoniae* isolates have all characteristics in common (i.e. resistance pheno- and genotype, biocide resistance genes, identical set of virulence associated genes, sequence type) including the same cgMLST type. They originated from different provinces and animals. *K. pneumoniae* is a known nosocomial pathogen with increasing multi-drug resistant rates and global dissemination [51]. Reports about *K. pneumoniae* ST610 are scarce. ST610 has two entries in the *K. pneumoniae* species complex database hosted at the Institute Pasteur (https://bigsdb.pasteur.fr/klebsiella/, last accessed 2023 May 30), whose corresponding isolates originated from a bovine mastitis in the USA and a human in Ireland. *K. pneumoniae* harboring *bla*_CTX−M−15_ has been observed in neighboring countries [52–54] but to our knowledge, there are no reports from Rwanda.

The *bla*_CTX−M−15_ gene was also carried by an ST163 *C. freundii* isolate, which is not a common clone. Until present, ST163 has three entries in PubMLST, from Canada (environment) and two human patients from China and Israel, respectively. Recently in Rwanda multi-drug resistant *C. freundii* harboring *bla*_CTX−M−15_, *bla*_OXA−10_, *bla*_OXA−1_, *bla*_TEM−1b_, and several non-β-lactamase resistance genes were described [41]. These isolates belonged to the ST328, a rarely observed lineage among *Citrobacter* spp [55, 56].

The majority of β-lactamase genes detected during the present study were predicted to be plasmid-borne. Plasmids play a significant role in the dissemination of ESBL genes [5]. Therefore, these findings could be of particular interest for public health in Rwanda. Several recent studies reported the occurrence of chromosomally located β-lactamase genes. Therefore, the differentiation between plasmid and chromosome localization of β-lactamase genes could be challenging, which is a limitation of the method.

## Conclusion

The present study contributes to the growing evidence that the presence of extended-spectrum cephalosporin-resistant Enterobacterales are currently part of the microbiome of ruminants in Rwanda. β-Lactamase genes frequently encoded on plasmids can easily be transferred horizontally. In addition, our data suggested that the spread of resistant clones could already have happened vertically through Rwanda. Considering the close contact between ruminants and humans in Rwanda, the dissemination of pandemic clones requires the joint and multisectoral action of human and veterinary medicine, at human-animal-environment interfaces. It is nowadays critical to establish national and global “One Health” surveillance programs of AMR to tackle the antibiotic-resistant crisis in human and veterinary medicine to ultimately protect the unique Rwandan wildlife, especially the population of endangered mountain gorillas (*Gorilla beringei* ssp. *beringei*) that plays a critical role in the lives of the people in Rwanda and in the resilience of the Rwandan natural ecosystem. Main limitations of this study rely in the challenges related to field work and data collection in resource-limited settings, difficult weather conditions, accessibility to farms, and communication with animal owners. Despite these inherent challenges, our results highlight the importance of AMR research in Africa.

## Electronic Supplementary Material

Below is the link to the electronic supplementary material.

Supplementary Material 1

Supplementary Material 2

Supplementary Material 3

## Data Availability

All data generated or analysed during this study are included in this published article [and its supplementary information files].

## Abbreviations

AMR: Antimicrobial resistance
aEPEC: Atypical enteropathogenic E. coli
BLAST: Basic Local Alignment Search Tool
BLDB: Beta-lactamase database
cgMLST: Core-genome Multilocus sequence typing
CH: fumC and fimH
ESBL: Extended-spectrum β-lactamases
ETEC: Enterotoxigenic E. coli
GLM: Generalized linear model
MLST: Multilocus sequence typing
NCBI: National Center for Biotechnology Information
QRDR: Quinolone resistance-determining regions
ST: Sequence type
STEC: Shiga toxin-producing E. coli
VAG: Virulence-associated genes
WGS: Whole-genome sequencing
WHO: World Health Organization
